# The Silent War of hMPV: Viral Interference with Host Immunity

**DOI:** 10.3390/biology15171444

**Published:** 2026-08-22

**Authors:** Grabiel J. García-Velázquez, Matías Moraga-Astete, Alison Sepúlveda-Pontigo, Karissa Chávez-Villacreses, Benjamín Díaz-López, Valeria Salazar-Montoya, Felipe Melo-González, Katina Schinnerling, Abel E. Vasquez, Claudio Cabello-Verrugio, Jorge A. Soto

**Affiliations:** 1 Center for Research on Pandemic Resilience, Faculty of Life Sciences and Institute of Public Health, Universidad Andres Bello, Santiago 8370186, Chile; grabiel.garcia95@gmail.com (G.J.G.-V.); matias.moragaastete@gmail.com (M.M.-A.); a.seplvedapontigo@uandresbello.edu (A.S.-P.); karissachavez8@gmail.com (K.C.-V.); b.dazlpez@uandresbello.edu (B.D.-L.); v.salazarmontoya@uandresbello.edu (V.S.-M.); felipe.melo@unab.cl (F.M.-G.); katina.schinnerling@unab.cl (K.S.); claudio.cabello@unab.cl (C.C.-V.); 2 Laboratory of Virology and Integrative Translational Immunology, Faculty of Life Sciences, Universidad Andrés Bello, Santiago 8370186, Chile; 3 Laboratory of Translational Immunology, Faculty of Life Sciences, Universidad Andrés Bello, Santiago 8370186, Chile; 4 School of Veterinary Medicine, Faculty of Medicine and Health Sciences, Universidad Mayor, Santiago 8580745, Chile; abel.vasquez@umayor.cl; 5 Center for Biomedicine, Universidad Mayor, Santiago 8580745, Chile; 6 Laboratory of Muscle Pathology, Fragility, and Aging, Faculty of Life Sciences, Universidad Andres Bello, Santiago 8370186, Chile

**Keywords:** human metapneumovirus, respiratory virus, antiviral response, innate and adaptive immune response, immune evasion

## Abstract

Human metapneumovirus (hMPV) is an important respiratory pathogen and a major cause of respiratory tract infections, particularly among vulnerable populations. Although hMPV was identified in 2001, it remains less extensively studied than other major respiratory viruses, such as influenza virus and respiratory syncytial virus (RSV), hindering the development of effective preventive and therapeutic strategies. This review examines the primary mechanisms by which hMPV evades host immune responses. The virus disrupts early innate immune signaling pathways, particularly those involved in the induction and signaling of antiviral interferons (IFNs) and modulates inflammatory responses. At the level of adaptive immunity, hMPV impairs T-cell activation and the development of long-lasting immunological memory, which may contribute to susceptibility to reinfection. The virus also alters the functions of several immune and structural cell types, including macrophages, dendritic cells, and respiratory epithelial cells. In addition, hMPV may modulate host microRNA expression to promote immune evasion and prolong infection. The recurrent nature of hMPV infections highlights the need to further investigate the immune-evasion mechanisms. Such research is essential for developing safer and more effective vaccines, antiviral agents, and immunomodulatory therapies.

## Simple Summary

Human metapneumovirus (hMPV) is a respiratory virus discovered about twenty years ago that causes frequent infections, which are especially serious in babies, older adults, and people with weak immune defenses. Even though the body tries to fight it, this virus has developed ways to hide from the immune system, blocking cells from making protective substances called interferons and preventing defense cells from “remembering” the virus for future infections. This review examines how different viral proteins block these defenses and how small molecules called microRNAs, which control gene activity, may also help the virus remain longer in the body. Understanding these mechanisms is essential for developing better vaccines and treatments against this virus, which continues to affect millions of people every year, particularly during colder months, and for which no specific prevention options are currently available.

## 1. Introduction

Human metapneumovirus (hMPV) is an enveloped, negative-sense, single-stranded RNA virus classified in the order *Mononegavirales*, family *Pneumoviridae*, and genus *Metapneumovirus* under the current taxonomy [1]. It was first identified in 2001 by van den Hoogen in the Netherlands while she was investigating pediatric patients presenting with acute respiratory tract infections of unknown etiology [2]. Although hMPV was first identified in the early 21st century, retrospective serological and molecular studies have demonstrated that hMPV had been circulating in humans for at least five decades before its discovery [3,4]. This delay in identification was mainly due to the virus’s inability to grow efficiently in standard cell culture systems and the lack of molecular diagnostic techniques for routine clinical use before the late 1990s [5]. The recognition of hMPV as a distinct pathogen has since prompted significant interest in its epidemiology, clinical spectrum, and public health impact. However, critical knowledge gaps remain regarding hMPV relative to what is known about other respiratory viruses, such as influenza and respiratory syncytial virus (RSV; recently renamed human orthopneumovirus).

hMPV is one of the leading viral causes of respiratory tract infections in infants, the elderly, immunocompromised patients and individuals with chronic conditions such as asthma and cancer [2,6,7]. Risk factors associated with an exacerbated immune response to hMPV infection in infants include asthma, preterm birth, and previous infections with other respiratory viruses such as RSV [8,9]. Regarding its epidemiology, hMPV circulates in the general population from winter through the end of spring [9,10]. hMPV remains underdiagnosed and understudied relative to other respiratory viruses such as influenza and RSV, despite its substantial global burden. This disparity in research attention is partly due to its often mild or self-limiting presentation in healthy individuals, which historically reduced its perceived clinical importance [2,4]. In addition, the absence of widely available and sensitive diagnostic tools in the early years after its discovery hindered detection and epidemiological characterization [7,11]. Finally, research priorities and funding have traditionally focused on pathogens with greater public health visibility or for which interventions are available, leaving hMPV comparatively neglected, despite evidence of severe disease in these populations. Interestingly, hMPV is now recognized as the cause of a significant proportion of acute respiratory tract infections (ARTIs) that were previously considered to be of unknown etiology, supporting early observations that hMPV had been circulating in the human population long before it was first isolated [2,12].

Two major genotypes of the virus exist, hMPV-A and hMPV-B, with further subdivisions: A1, A2 (including the A2a, A2b, and A2c lineages), B1, and B2 (comprising the B2a and B2b lineages) [13,14]. These genotypes differ mainly in the nucleotide and amino acid sequences of their surface glycoproteins, with the attachment glycoprotein G showing substantially greater genetic variability than the fusion protein F, which is highly conserved between hMPV-A and hMPV-B [15].

Various studies have focused on the correlation between hMPV subtypes and clinical characteristics in infected patients. Vicente et al. found that the clinical severity of hMPV A infections was higher than that of hMPV B infections in a pediatric cohort [16]. Matsuzaki et al. indicated that laryngitis was more associated with hMPV B1 infections, whereas wheezing was more prevalent with hMPV B1 and B2 infections [17,18]. However, several large cohort studies have found no significant clinical differences between genotypes A and B, including a prospective study in hospitalized children in which no clinical differences were observed between genotypes A and B [19]. Experimental work in animal models has further supported the absence of a consistent association between genotype and severity: a study in a murine model found that, although genotype B exhibited a competitive replication advantage over genotype A, it did not cause more severe disease as measured by body weight, airway hyperreactivity, and lung pathology [20].

The hMPV genome encodes nine proteins, including those essential for replication and modulation of the host immune response [21]. The viral replication cycle occurs in the cytoplasm, where the viral RNA-dependent RNA polymerase replicates and transcribes the genome [13].

Although hMPV triggers both innate and adaptive immune responses, it has evolved several mechanisms to evade immune recognition and clearance. The virus interferes with type I and III interferon (IFN) signaling pathways involved in innate immunity. This interference prevents the activation of sensors such as retinoic acid-inducible gene I (RIG-I) and helicase melanoma differentiation-associated gene 5 (MDA5) [22].

Moreover, viral proteins such as matrix 2-2 protein (M2-2), attachment glycoprotein (G), and small hydrophobic protein (SH) have been associated with the modulation of the innate immune response through their effects on key molecules, including NF-κB, interferon regulatory factor 3 (IRF3), and interferon regulatory factor 7 (IRF7). As a result, the production of IFNs and proinflammatory cytokines is reduced [23,24,25]. hMPV also impairs adaptive immunity by inhibiting T-cell activation and the development of long-lasting immunological memory [26]. Additionally, Céspedes et al. in 2013 suggested that T cells are not activated during hMPV infection; instead, hMPV induces T-cell anergy or immunosuppression [27]. Studying these immune evasion mechanisms is essential not only for understanding the pathogenesis of hMPV infection but also for developing effective prevention and treatment strategies [27]. The objective of this paper is to examine the molecular and cellular mechanisms that enable hMPV to evade the host immune response. The primary focus will be on key viral proteins and their effects on disease severity and treatment efficacy.

## 2. Literature Search Methodology

This narrative review was based on a comprehensive literature search of the PubMed/MEDLINE, Scopus, and Web of Science databases. Searches were conducted using combinations of the following terms: human metapneumovirus, hMPV, immune evasion, innate immunity AND hMPV, adaptive immunity AND hMPV, interferon signaling AND respiratory virus, dendritic cells AND hMPV, T cell exhaustion AND respiratory virus, and microRNA AND hMPV. No strict publication date restriction was applied, as both recent studies on hMPV and classic publications on general immunology were included as necessary to contextualize the mechanisms of innate and adaptive immune responses discussed.

Eligible studies included peer-reviewed original research articles and review articles published in English that addressed the molecular, cellular, or immunological mechanisms by which hMPV evades host innate and adaptive immune responses, as well as foundational studies on general immune system biology relevant to this context. Studies involving related respiratory viruses (e.g., RSV, influenza, SARS-CoV-2) were included when relevant for comparative purposes, particularly regarding shared or conserved immune evasion strategies such as microRNA modulation. Editorials, conference abstracts without original data, and non-English publications were excluded.

Additional relevant publications were identified through manual review of the reference lists of selected articles. Given the narrative nature of this review, the literature was synthesized qualitatively, with emphasis on studies that have advanced current understanding of hMPV pathogenesis and that may inform future vaccine and antiviral development strategies.

## 3. Epidemiology of hMPV

Acute respiratory tract infections (ARTIs) are a major cause of morbidity and mortality worldwide and are considered a significant global public health threat [28,29]. In 2001, a research team in the Netherlands identified hMPV in nasopharyngeal specimens from children suffering from acute respiratory tract infections, utilizing direct electron microscopy [2]. Together with other respiratory viruses, including influenza viruses, RSV, and coronaviruses, hMPV contributes to the global burden of ARTIs [28]. Since its initial identification, hMPV has been reported worldwide and represents a significant cause of ARTIs, largely attributable to insufficient prevention and control mechanisms [30,31].

hMPV is primarily transmitted through respiratory droplets expelled by infected individuals. While hMPV infections are often mild and self-limiting, they may follow a complicated clinical course in vulnerable populations [32]. Symptoms resemble those of other respiratory viral infections and range from mild (cough, rhinorrhea, and fever) to more severe conditions (bronchiolitis and pneumonia) [33]. In severe cases, the patient’s clinical condition may deteriorate, necessitating urgent medical intervention [2,19,34]. The epidemiology of hMPV follows a recurring seasonal pattern, with prevalence increasing during colder months, particularly among these vulnerable populations.

hMPV infection accounts for nearly one million outpatient clinic visits for ARTI-related symptoms annually, and the disease poses a substantial threat to the health of over 86% of children under five years of age worldwide [9,28,35]. This virus poses a substantial burden on healthcare systems worldwide. For instance, this virus causes over 20,000 hospitalizations in the US each year, and the cost per infected patient ranges from USD 3850 to USD 9946 [9,30]. Database records indicate that hMPV has been extensively prevalent in various regions of China. Although the prevalence of hMPV is comparatively lower than certain ARTI-related viruses, such as HRV and RSV, the public health threats it presents cannot be overlooked [28,36].

Before the COVID-19 pandemic, reported hMPV circulation was limited in Latin America, particularly in Chile, where respiratory virus surveillance is well established. However, there was a significant increase in 2022 and 2023, with the most considerable rise occurring in 2023. This increase was likely associated with the relaxation of COVID-19-related public health measures, which had previously limited viral transmission. The number of reported cases rose to over 5559 in 2022 and 6455 in 2023, and the growth was even more pronounced in 2024 [37]. The virus’s global spread and its propensity to cause respiratory infections ranging from mild to severe highlight its importance as a new pathogen. Although its clinical signs match those of other respiratory viruses, precise diagnostic identification of hMPV facilitates a more accurate evaluation of its public health impact. This epidemiological context is particularly relevant when examining the mechanism by which hMPV manifests its pathogenicity.

## 4. Viral Cycle of hMPV

**Figure 1:**
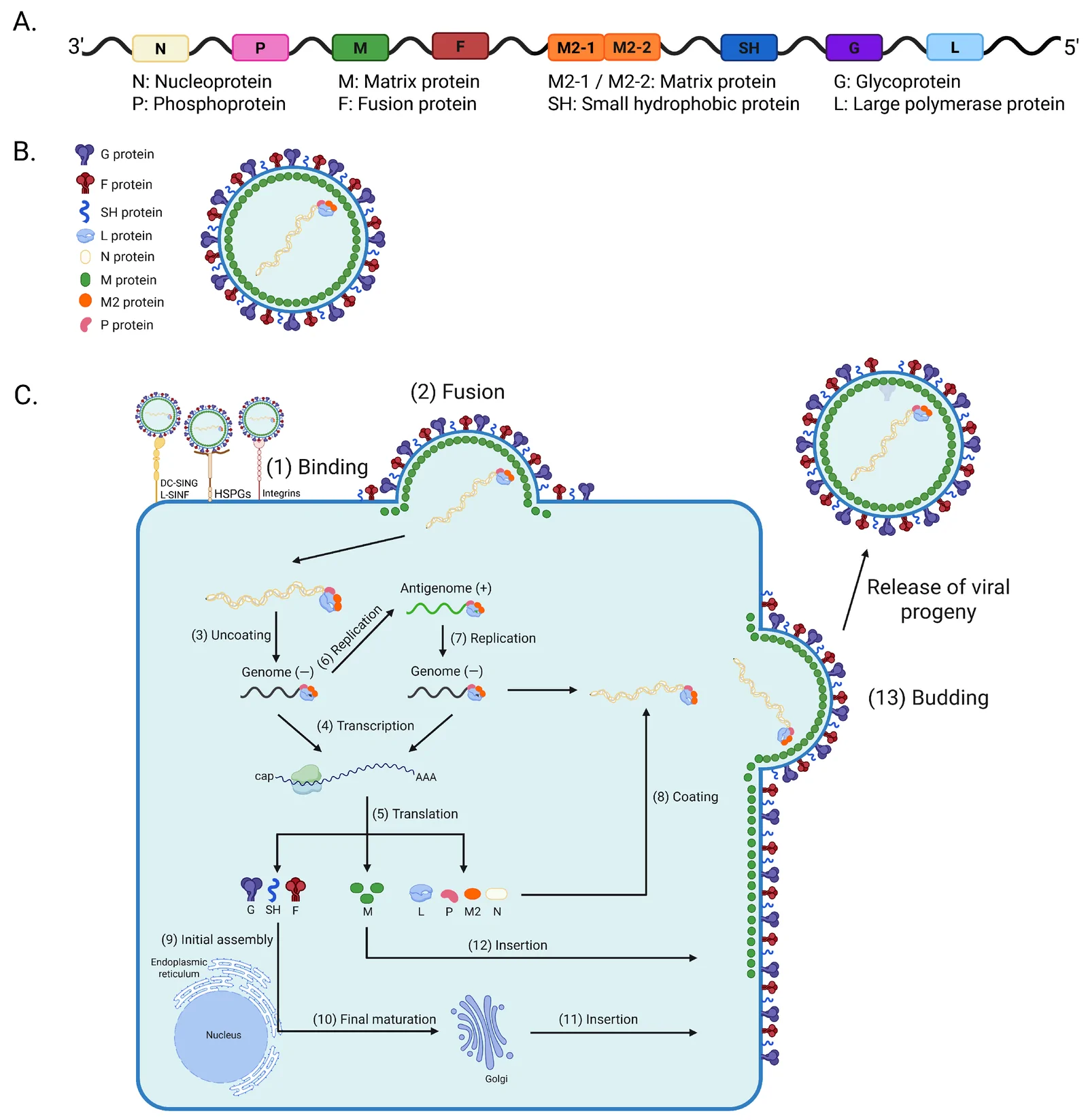
Genome, structure, and viral cycle of hMPV. (**A**) Representation of the viral genome of hMPV. (**B**) Basic structure of an hMPV virion. (**C**) Schematic representation of the viral replication cycle of hMPV. The virus attaches to respiratory epithelial cells via the G protein and enters through F protein-mediated fusion (steps 1–3); the viral RNA-dependent RNA polymerase then transcribes and replicates the genome in the host cell cytoplasm (steps 4–8); and finally, the late events of the cycle consist of the maturation, assembly, and release of new virions through a budding mechanism (steps 9–13).

hMPV possesses a single-stranded negative-sense RNA genome with eight genes encoding nine proteins. Gene organization follows a 3′ to 5′ order according to its abundance in the viral particle (Figure 1A). Specifically, the *N* gene encodes the viral nucleoprotein, the *P* gene encodes the phosphoprotein, the *M* gene encodes the matrix protein, the *F* gene encodes the viral fusion glycoprotein, the *M2* gene encodes two proteins, M2.1 and M2.2, the *SH* gene encodes the viral small hydrophobic protein, the *G* gene encodes the G glycoprotein, and the *L* gene encodes the large polymerase protein [34,38,39]. The viral structure and replicative cycle are shown in Figure 1B,C.

The replication cycle of hMPV begins when specific receptors on the host cell recognize the surface proteins of the virion. The G glycoprotein interacts explicitly with heparan sulfate proteoglycans (HSPGs) as well as with the receptors DC-SIGN (dendritic cell-specific intercellular adhesion molecule-3-grabbing nonintegrin) and L-SIGN (liver/lymph node-specific intercellular adhesion molecule-3-grabbing nonintegrin), facilitating viral attachment to epithelial, dendritic, and endothelial cells [40]. The F protein helps the viral envelope fuse with the plasma membrane, and this process is made easier by its interaction with integrins [13]. The viral genome, which is negative-sense, single-stranded RNA, is released into the cytoplasm and uncoated when the N protein breaks down. The viral RNA-dependent RNA polymerase, along with its cofactor P protein, locates the 3′ end of the genome and initiates transcription. This produces monocistronic, positive-sense mRNAs that have a cap at the 5′ end and polyadenylation at the 3′ end. The ribosomes in the host cell use these transcripts to synthesize viral proteins. The M2-1 protein is an elongation factor that the P protein recruits to attach to the new viral RNA, stabilizing it and facilitating processing. As the N protein builds up in the cytoplasm, the viral machinery shifts from transcription to replication, producing a positive-sense antigenome that serves as a template for creating new negative-sense genomic RNA. The N protein protects both antigenomes and genomes by encapsulating them and incorporating them into new viral particles. The endoplasmic reticulum synthesizes the envelope proteins F, G, and SH. The Golgi apparatus modifies them, and then they are sent to the plasma membrane. Simultaneously, the M protein accumulates at the membrane and recruits the nucleocapsid to form additional virions, which are then released through budding [9,40] (Figure 1C).

The N, L, and P proteins work together to create the viral replication complex. The gene order of hMPV is different from that of hRSV, and the virus also does not have the non-structural proteins NS1 and NS2 [2,12]. The lipid envelope contains three transmembrane surface glycoproteins: F, G, and SH. The G protein is necessary for the virion to attach to the host cell. The F protein helps the viral and host cell membranes fuse together. The precise role of the SH protein is still not clear. The F protein sequence is comparatively well-conserved among various hMPV genotypes, in contrast to the G and SH proteins, which exhibit greater variability [12,15,41,42,43]. Viral gene products are characterized in Table 1, as previously reported [33,38].

## 5. Viral Proteins and Their Contribution to the Immune Response

The roles of some viral proteins in hMPV evasion remain under investigation. The G protein has been characterized as a highly glycosylated type II mucin-like glycoprotein with both N-linked and O-linked sugars [41,42,44,45]. Extensive glycosylation may contribute to interference with protective immunity, as it has been reported for the G protein of RSV, and may facilitate viral evasion of host immune responses [44,46]. HSPGs are necessary for hMPV to adhere to cell surfaces because they are found in many places and can bind to viral particles. The G and F proteins enable the virus to infect host cells more effectively and evade immune monitoring by altering how the host’s immune system functions [13].

The G protein can suppress the IFN-I response both in vitro and in vivo [47,48]. The latter investigation demonstrated that the G protein is linked to the recruitment of neutrophils into the alveolar space in the lungs of mice infected with hMPV. The authors argue that this phenomenon results from the suppression of the IFN response, identifying significant alterations in molecules implicated in neutrophil recruitment, including C-C chemokine ligand 3 (CCL3), C-C chemokine ligand 4 (CCL4), Vascular Endothelial Growth Factor (VEGF), Tumor Necrosis Factor (TNF), interleukin 17 (IL-17), and CXCL2 [25,48]. In addition, the G protein directly interacts with the cytoplasmic sensor RIG-I, preventing it from connecting with MAVS. This connection prevents the formation of the mitochondrial signaling complex needed to activate the transcription factors IRF-3 and NF-κB. This leads to reduced production of type I IFNs and other proinflammatory cytokines [47]. Mutations in the Glu2 and Val3 residues of the G protein eliminate this inhibitory action, underscoring their significance in immunological regulation [49].

In monocyte-derived dendritic cells (moDCs), the G protein suppresses Toll-like receptor 4 (TLR4)-dependent signaling. Kolli et al. infected moDCs with wild-type recombinant hMPV-A (rhMPV-WT) or recombinant hMPV-A deficient in G protein production (rhMPV-ΔG). Twenty-four hours after infection, the cells were washed and treated with LPS. The supernatants were then collected to determine how much cytokine and chemokine was produced. They discovered that LPS-induced production of cytokines, including IL-6, IL-8, IL-10, IFN-γ, and TNF-α, as well as chemokines such as RANTES and MIP-1β, was significantly decreased by infection with rhMPV-WT, but not by rhMPV-ΔG. This indicates that the G protein may block TLR4-dependent signaling during hMPV infection. Similarly, pretreatment of moDCs with purified G protein reduced LPS-induced proinflammatory mediators in a dose-dependent manner. Cells treated with only LPS generated substantial quantities of proinflammatory mediators. These findings demonstrate that hMPV G protein efficiently obstructs TLR4-dependent cellular signaling [50].

The G protein also plays a significant role in avoiding adaptive immunity. The G and SH glycoproteins have been demonstrated to impede macropinocytosis in human dendritic cells, thereby reducing viral uptake and the subsequent activation of CD4^+^ T cells. Recombinant strains devoid of these glycoproteins lead to increased CD4^+^ T cell proliferation and more robust Th1-type responses, highlighting their inhibitory function in adaptive immunity [51]. Understanding how the G protein of the hMPV-A strain affects the immune system has led to the creation of weakened virus strains that could be used as vaccines. Research in mouse models indicates that the deletion of the G protein augments the activation of immune cells, such as neutrophils, dendritic cells, NK cells, and B cells, without inducing clinical disease. These results endorse the utilization of G-deficient hMPV strains in the formulation of live attenuated vaccines [52].

Conversely, several pneumovirus SH proteins generate more substantially glycosylated variants, but this phenomenon has yet to be conclusively seen in paramyxoviruses [38,45,53]. Identified SH proteins are type I or II transmembrane (TM) proteins characterized by a single-pass TM domain. The functions of SH proteins remain largely uncharacterized, but some of them have viroporin activity, which makes the membranes of infected cells or cells that produce SH protein more permeable [38,54].

Specifically, the hMPV SH protein has been linked to the suppression of NF-κB-dependent gene expression, with NF-κB being a transcription factor crucial for the activation of proinflammatory cytokines, including IL-6 and IL-8 [23]. In fact, removing SH from the hMPV-A strain (hMPV-ΔSH) increases the likelihood of these cytokines being produced, suggesting that SH has an immunomodulatory role by limiting NF-κB activation. In airway epithelial cells and mouse models, infection with hMPV-ΔSH induced increased secretion of IL-6 and IL-8 relative to the wild-type virus [23,38]. At the molecular level, the SH protein was found to stop the transcription of NF-κB-dependent genes by blocking the TNF-α-induced activation of the IL-8 promoter. This effect does not seem to be caused by a change in NF-κB nuclear translocation, since both p65 and p50 translocate to the nucleus at the same rate following infection with either hMPV-WT or ΔSH. Instead, the SH protein prevents significant posttranslational changes to NF-κB, such as phosphorylation at Ser276 and Ser536, and acetylation at Lys310 of the p65 subunit [23]. These changes are necessary for NF-κB to function entirely as a transcription factor. These results show that the hMPV SH protein is crucial for controlling the innate immune response by specifically altering NF-κB transcriptional activity. In contrast to other paramyxoviruses, which suppress NF-κB nuclear translocation directly, the hMPV mechanism appears to differ, primarily by altering phosphorylation and acetylation of transcription factors involved in signaling pathways [23,38].

Moreover, infection with a recombinant hMPV-A strain deficient in SH induced phosphorylation of the transcriptional activator 1 (STAT1) at levels comparable to those in untreated cells [55]. hMPV-A infection facilitated the proteasomal degradation of JAK1 and TYK2, while the underlying mechanism was not elucidated [56]. Since hMPV infection has been associated with the suppression of an alternative JAK-STAT signaling pathway involving IL-6, a common mechanism involving SH is conceivable. The results of the study by Brynes et al. validated the claim that SH can block IFN and IL-6 signaling by reducing JAK proteins, which are involved in the signaling of these molecules. SH was both necessary (during infection) and appropriate (under transfection conditions) to obstruct IL-6 signaling [57]. In vitro, hMPV-A infection activates the NLRP3 inflammasome in an SH-dependent manner, akin to hRSV SH [38,58]. The same work demonstrated that suppressing NLRP3 or deleting IL-1β in vivo significantly reduced mortality in high-inoculum hMPV mouse infections, indicating a potential role for hMPV SH in facilitating pathogenesis. All sequenced clinical isolates of hMPV possess an SH gene; nonetheless, viral passage in cultured cells results in significant frameshift and point mutations, indicating that the in vivo functions of SH are non-essential in vitro [38,59]. Conversely, de Graaf et al. could not ascertain any role of SH in viral replication or the immune response in vitro within host cells utilizing an hMPV mutant deficient in SH [12,60].

Another envelope protein of hMPV is the fusion protein (F), which is highly conserved among hMPV subgroups and shares a similar structural topology and approximately 30% amino acid sequence homology with the fusion protein of RSV. hMPV F is a class I fusion glycoprotein synthesized as an inactive precursor (F0) that must undergo proteolytic cleavage to become fusion-competent. Proteolytic cleavage generates two disulfide-linked subunits, with F2 at the N-terminus and F1 at the C-terminus, which assemble into a homotrimer. Cleavage occurs at a monobasic cleavage site immediately upstream of the hydrophobic fusion peptide. The prefusion conformation of hMPV F is metastable and undergoes a conformational rearrangement to the postfusion state during the membrane fusion process [61,62].

This protein is the primary target of neutralizing antibodies, making it a critical antigen for vaccine design. The antigenic surface is organized according to the same nomenclature established for the RSV F protein, encompassing sites Ø through V. Sites Ø, V, and the more recently described site VI are exclusively exposed in the prefusion conformation, whereas sites I to IV are shared between the pre- and postfusion states. Interestingly, unlike RSV, in which prefusion-specific epitopes elicit disproportionately potent neutralizing antibody responses, both the prefusion and postfusion conformations of hMPV F have been reported to induce comparable neutralizing antibody titers. Similarly, independent serum depletion and murine immunization studies found no significant antigenic differences between the two conformations of hMPV F [63].

Antigenic site III has emerged as a particularly relevant target. It is located near site I and is recognized by monoclonal antibodies such as MPE8 and 25P13, which typically bind at the interface between two adjacent F protomers. Given that RSV and hMPV share only 30–35% amino acid sequence identity in their F proteins, the isolation of broadly neutralizing antibodies capable of cross-neutralizing both viruses through site III was unexpected [64]. This class of cross-reactive antibodies is characteristically encoded by the IGHV1-23 heavy-chain gene and exhibits restricted light-chain variable gene usage [65]. A human antibody targeting the hMPV-specific region near site III, termed MPV364, has also been described. Although it does not cross-react with the RSV F protein, it substantially reduced viral replication in the lungs of infected mice, highlighting its therapeutic potential [64]. Antigenic site IV is likewise recognized by cross-neutralizing antibodies, including 101F, 54G10, and 17E10. Notably, 54G10 binds near site IV on the RSV F protein and reduces lung viral titers of both hMPV and RSV in vivo. More recently, antibodies targeting site V, which is specific to the prefusion conformation, have also been characterized as potent cross-neutralizers of both viruses [63].

Alongside the investigated G, SH and F proteins, additional viral proteins have been identified as facilitators of hMPV immune evasion. M2-1 and M2-2 are encoded in matrix 2 (M2) mRNA. The translation of the M2-2 protein likely commences independently of the M2-1 ORF, as evidenced by the robust production of M2-2 in cells infected with hMPV-A containing a translation-silenced M2-1 ORF [66,67]. The viral M2 proteins (M2-1 and M2-2) are also crucial for polymerase activity. M2-1 is an antitermination factor necessary for viral replication, while M2-2 appears to control the switch from positive antigenomic to negative genomic RNA during late-stage replication [68,69].

The M2-2 protein inhibits MAVS, thereby facilitating hMPV immune evasion and suppressing MAVS-dependent cellular responses [25,70]. MAVS is necessary for the activation of NF-κB and IRF3. If this protein is blocked, viral replication and cell death increase. If it is overexpressed, the synthesis of IFN (activated by NF-κB and IRF3) increases, leading to an antiviral state [71]. PDZ-binding motifs in an immune-inhibitory region of M2-2 enable M2-2 to evade the immune system. hMPV activates NF-κB and IRF3 by using tumor necrosis factor receptor-associated factor (TRAF)5, TRAF6, and TRAF3, which are recruited by MAVS. When M2-2 is suppressed, the immunological response that relies on MAVS is also stopped. This is because M2-2 uses its PDZ motifs to prevent the interaction between MAVS and its downstream TRAF signaling [25,72]. Ren et al. also found that dendritic cells infected with an hMPV-A mutant lacking the M2-2 protein produced more MyD88-dependent genes, such as cytokines, chemokines, and type I IFNs [73]. More specifically, M2-2 inhibits the TLR7/9-dependent signaling pathway by preventing IRF7 homodimerization through partial suppression of IRF7 phosphorylation [12,24]. The viral M2-2 protein has been associated with the downregulation of antigen presentation through major histocompatibility complex (MHC) class I molecules. This process diminishes the host immune system’s capacity to identify and eliminate infected cells in numerous viral infections [74,75]. Choi et al. and other researchers used a reverse-engineered hMPV virus with modifications in the M2-2 PDZ-binding motif to infect mice. This caused increased dendritic cell (DC) maturation and recruited more DCs and T cells to the lungs. These in vivo findings underscore the potential for altering M2-2 PDZ-binding motifs to enhance mucosal innate immunity during hMPV infection and suggest a promising alternative candidate for an hMPV vaccine that confers protection against hMPV infection [76].

hMPV, like all pneumoviruses, has the zinc-binding protein M2-1, which is a critical part of RNA synthesis because it prevents the termination of viral RNA transcription. Its activity is crucial for hMPV proliferation and pathogenesis; yet, it has not been explicitly linked to the modulation of the host immune response [77]. Some researchers have proposed that changes in this zinc-binding protein can change how easily hMPV spreads, making it less likely to cause infection in cotton rats [68].

To date, evidence indicates that hMPV evades host immune responses through multiple mechanisms involving viral proteins that impair the host’s ability to recognize and respond to viral infections. The G protein is particularly relevant because it can inhibit type I IFNs by interfering with signaling through receptors like TLR4 and RIG-I. This breaks one of the host’s initial lines of immunological protection. The M2-2 protein is also essential because it stops intracellular signaling pathways that cause IFN activation, which makes interferon-stimulated genes less active. Researchers are still working to determine precisely what the SH protein does, although it has been linked to transcription factor NF-κB activity. Conversely, proteins like F and P, which are crucial for viral replication and entry, lack direct evidence of immune evasion capabilities; yet their indirect role in pathogenesis cannot be dismissed entirely. These mechanisms facilitate hMPV persistence and replication within the host by allowing the virus to evade key components of the innate immune response, thereby hindering infection control.

## 6. Non-Coding RNA Regulation During hMPV Infection

Recent evidence suggests that hMPV infection is associated with extensive post-transcriptional reprogramming of host cells through the modulation of microRNA (miRNA) expression [78]. miRNAs are short, non-coding RNAs that control gene expression through translational repression or mRNA degradation. They are increasingly recognized as critical regulators of antiviral immunity, inflammatory signaling, apoptosis, and immune cell differentiation [79]. In respiratory viral infections, modulation of host miRNA profiles has emerged as an important strategy by which viruses manipulate intracellular antiviral responses and establish a permissive environment for replication and persistence [80].

The specific molecular mechanism by which hMPV modulates miRNA expression remains uncharacterized. miRNA biogenesis requires sequential cleavage by two RNase III enzymes: Drosha in the nucleus (which cleaves pri-miRNA to pre-miRNA) and Dicer in the cytoplasm (which cleaves pre-miRNA to mature miRNA), followed by loading onto Argonaute proteins to form the functional RISC complex [81,82]. Direct evidence demonstrating that hMPV proteins target or degrade these canonical biogenesis factors has not yet been established. In contrast, related respiratory viruses such as SARS-CoV-2 and RSV do have strategies that target the miRNA biogenesis machinery [83,84,85], but whether hMPV uses similar or distinct mechanisms is still unknown.

What is already experimentally demonstrated is the induction of a coordinated and reproducible pattern of miRNA expression by hMPV infection; this pattern affects IFN signaling, inflammatory regulation, antigen presentation and cell survival [78]. These phenotypic changes may result from multiple potential mechanisms, which may include direct targeting of biogenesis factors, sequestration of mature miRNAs, recruitment of viral nucleases or indirect transcriptional reprogramming, but the operative mechanism(s) by which hMPV alters infected cells have not been established.

### 6.1. miRNA Modulation in Respiratory Epithelial Cells

Respiratory epithelial cells are the primary targets of hMPV infection and constitute one of the first cellular barriers involved in the initiation of antiviral immune responses [86]. In addition to producing type I and type III IFNs, epithelial cells actively participate in the secretion of cytokines and chemokines that regulate immune cell recruitment and local inflammatory responses [80]. Recent evidence suggests that hMPV infection induces significant alterations in host microRNA (miRNA) expression within epithelial cells, thereby modulating intracellular pathways associated with antiviral defense, apoptosis, and inflammatory signaling [78].

During hMPV infection of A549 epithelial cells, differential expression of several miRNAs has been identified, including upregulation of let-7f and miR-452, together with downregulation of miR-374a and miR-192 [78]. These findings suggest that hMPV may induce a coordinated post-transcriptional regulatory program capable of modulating multiple pathways involved in viral replication and immune evasion.

Among these miRNAs, members of the let-7 family have been extensively associated with negative regulation of inflammatory signaling pathways, including suppression of Toll-like receptor (TLR)-mediated responses and attenuation of NF-κB activation [87]. Additionally, let-7 family members have been implicated in the regulation of IL-6 production and maintenance of immune homeostasis during viral infections [88]. Consequently, increased expression of let-7f during hMPV infection may contribute to the balanced regulation of antiviral signaling pathways downstream of pattern-recognition receptors such as TLR3 and RIG-I, thereby limiting excessive activation of antiviral responses while preserving epithelial-cell viability [87]. Furthermore, since the let-7 family has also been associated with the regulation of apoptosis and cellular metabolic adaptation [89], hMPV-induced upregulation of let-7f may additionally favor prolonged survival of infected cells.

Similarly, increased expression of miR-452 may contribute to the establishment of a cellular environment favorable for viral replication. Previous studies demonstrate that miR-452 is significantly upregulated in pathological conditions and functions as a promoter of cell proliferation and survival by targeting cell-cycle inhibitors (such as CDKN1B/p27) and suppressing apoptosis-associated pathways. Enhanced miR-452 expression also promotes activation of pro-survival signaling cascades including EGFR and AKT pathways. During hMPV infection of epithelial cells, upregulation of miR-452 could similarly suppress apoptosis and extend the proliferative lifespan of infected cells, thereby facilitating persistent viral replication [90,91].

In contrast, hMPV infection has also been associated with downregulation of miR-374a and miR-192, both of which have been implicated in the regulation of inflammatory signaling and antiviral cellular homeostasis. miR-374a has been described as a negative regulator of NF-κB-associated inflammatory pathways and cytokine production [92]. Consequently, reduced expression of miR-374a during hMPV infection may contribute to dysregulated inflammatory responses and enhanced pulmonary immunopathology, which are characteristic features observed during severe respiratory viral infections [93]. Likewise, miR-192 has been associated with the regulation of p53-dependent pathways, the induction of apoptosis, and antiviral cellular responses [94]. Since apoptosis constitutes an important antiviral mechanism limiting viral replication, suppression of miR-192 during hMPV infection may impair apoptosis-mediated clearance of infected cells, thereby promoting prolonged cell survival and facilitating viral persistence.

Collectively, these findings suggest that hMPV-induced modulation of epithelial-cell miRNAs may contribute to the attenuation of antiviral signaling pathways, dysregulation of inflammatory responses, and prolonged survival of infected cells, thereby creating a permissive intracellular environment that favors viral replication and persistence within the respiratory tract.

### 6.2. miRNA Regulation in Dendritic Cells and Antiviral Immunity

In addition, to epithelial cells, hMPV infection has also been shown to alter miRNA expression in moDCs, suggesting that post-transcriptional regulation may additionally contribute to the modulation of innate and adaptive immune responses [78]. Dendritic cells play a central role in antigen presentation, interferon production, and activation of antiviral T-cell responses [26]; therefore, alterations in miRNA expression within these cells may significantly affect the development of protective immunity during hMPV infection.

Among the miRNAs differentially expressed in hMPV-infected moDCs, increased expression of miR-182-5p and miR-4634 has been reported [78]. Although the precise mechanisms associated with these miRNAs remain incompletely characterized, current evidence suggests that they may participate in the regulation of interferon-mediated antiviral signaling, dendritic cell maturation, and T-cell activation.

miR-4634 has been proposed as a potential negative regulator of type I IFNs responses during hMPV-A infection. Since type I IFNs constitute one of the major antiviral defense mechanisms induced during the early stages of viral infection, attenuation of interferon-associated signaling pathways may significantly favor viral replication. Although the direct molecular targets of miR-4634 during hMPV infection remain unknown, increased expression of this miRNA may interfere with signaling pathways associated with interferon-stimulated gene (ISG) activation, thereby reducing the establishment of an effective intracellular antiviral state [80]. Consequently, upregulation of miR-4634 may facilitate viral replication and persistence within infected cells while simultaneously impairing dendritic cell antiviral functionality.

Similarly, miR-182-5p has been associated with the regulation of immune cell differentiation, dendritic cell activation, and T-cell responses through pathways involving FOXO1-associated signaling networks [95]. FOXO1 plays an important role in antigen presentation, co-stimulatory molecule expression, and activation of antiviral T-cell responses [96]. Therefore, altered expression of miR-182-5p during hMPV infection may contribute to impaired dendritic cell maturation and reduced T-cell priming capacity. Additionally, dysregulation of miR-182-associated pathways has been linked to dysfunctional T-cell differentiation and exhaustion-associated phenotypes during chronic viral infections [97], raising the possibility that hMPV-induced miRNA modulation may contribute to the incomplete and short-lived protective immunity characteristic of recurrent hMPV infections.

Collectively, these observations suggest that hMPV-induced modulation of miRNA expression in moDCs may contribute not only to the suppression of interferon-mediated antiviral responses, but also to impaired antigen presentation and dysfunctional adaptive immune activation. Such coordinated post-transcriptional regulation may represent an important mechanism by which hMPV interferes with the establishment of effective long-term immunological memory, thereby facilitating recurrent infections throughout life.

### 6.3. Integrated Role of miRNAs in Immune Evasion and Viral Persistence

Although the precise targets and mechanisms of many hMPV-associated miRNAs remain incompletely understood, current evidence supports the hypothesis that hMPV induces a coordinated miRNA-mediated reprogramming of host immune responses. Upregulated miRNAs, including miR-4634, miR-182-5p, let-7f, and miR-452, may collectively attenuate antiviral IFN signaling, alter antigen presentation, regulate inflammatory pathways, and prolong the survival of infected cells. In parallel, downregulation of miR-192 and miR-374a [78] may remove critical antiviral and anti-inflammatory regulatory checkpoints, thereby promoting inflammatory dysregulation and inefficient viral clearance.

Rather than acting independently, these miRNAs likely function as interconnected components of a broader regulatory network that reshapes both innate and adaptive immunity during hMPV infection. Such coordinated post-transcriptional immune modulation may help explain several hallmark characteristics of hMPV pathogenesis, including incomplete sterilizing immunity, weak immunological memory, prolonged airway inflammation, and recurrent infections throughout life.

Furthermore, growing evidence from related respiratory viruses, including RSV, influenza virus, and SARS-CoV-2, suggests that manipulation of host miRNA pathways represents a conserved viral strategy for immune evasion and persistence. RSV, influenza, and SARS-CoV-2 each employ distinct but functionally similar mechanisms of host miRNA modulation to suppress IFN responses and promote viral replication. Consequently, further characterization of hMPV-associated miRNA signatures may not only improve understanding of viral pathogenesis but also identify novel biomarkers and therapeutic targets for future antiviral interventions [80,98].

**Figure 2:**
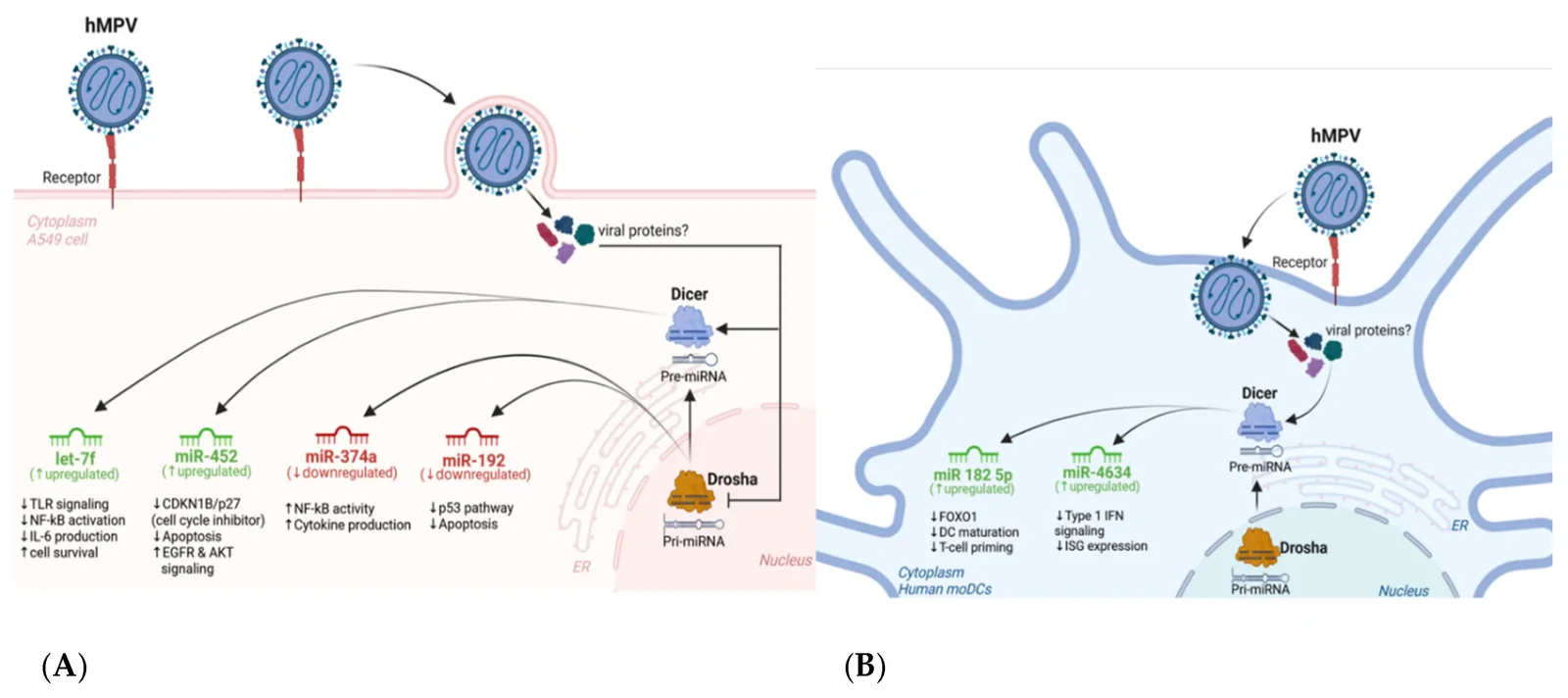
Coordinated alteration of microRNA biogenesis and expression during hMPV infection in host cells [78]. (**A**) In respiratory epithelial cells (A549), hMPV infection induces changes in the expression of multiple microRNAs (miRNAs) associated with modulation of cell survival, regulation of apoptosis, and antiviral responses. Upregulation of let-7f and miR-452 promotes viral persistence through attenuation of inflammatory responses and inhibition of pro-apoptotic signals, while downregulation of miR-374a and miR-192 compromises antiviral defenses. (**B**) In moDCs, hMPV infection results in selective upregulation of miR-182-5p and miR-4634, whose elevated expression interferes with dendritic cell maturation and T cell activation by suppressing critical signaling pathways such as FOXO1 and type I interferon-mediated responses. Question marks indicate uncertainty regarding the exact mechanisms by which hMPV alters miRNA expression through potential interference with the miRNA processing machinery (Drosha and Dicer). These coordinated changes in miRNA expression represent a sophisticated viral strategy that contributes to immune evasion, viral persistence, and a propensity for recurrent reinfections.

In summary, the studies described above demonstrate that hMPV-regulated miRNAs play important roles in modulating host cellular responses during infection. As summarized in Figure 2, several cellular signaling, immune response, and survival pathways are affected by these miRNAs. However, unlike other respiratory viruses, the molecular mechanisms by which hMPV manipulates the canonical miRNA biogenesis pathway remain largely unknown. It is still unclear whether specific hMPV proteins directly interfere with key components of the canonical miRNA processing machinery, such as Drosha or Dicer.

## 7. Interferon and hMPV

Interferons are cytokines that are categorized into three groups: Type I, II, and III IFNs. Their functions are modulated by the JAK-STAT pathway [26]. The IFN-I receptor complex (IFNAR) is found in all nucleated cell types, while IFN-II and IFN-III receptor complexes (IFNLR and IFNGR) are primarily located in epithelial and immune cells, respectively [9,99]. For a virus to successfully infect a host, it must employ effective strategies to bypass or attack the host’s innate immune defenses, including the production of type I IFNs by infected cells. Type I IFNs are the initial line of defense against viral infections and play an essential role in stopping the spread of viruses by activating hundreds of distinct IFN-stimulated genes (ISGs). Some ISGs are viral sensors that are commonly overexpressed to make it easier to detect viruses infecting cells. Other ISGs code for proteins that can assist in desensitizing the IFN activation pathway [9,100,101]. These genes induce an antiviral state by inhibiting fundamental activities in adjacent cells. IFN receptors (IFNRs) are present on target cells, including both innate and adaptive immune cells, facilitating the destruction of infected cells by enhancing both antigen-dependent and -independent cytotoxicity [66,102,103,104]. Viruses can impede intracellular sensing mechanisms that stimulate IFN gene production, specifically RIG-I-like receptors and the cGAS–STING axis, or hinder signaling induced by IFNs [105].

**Figure 3:**
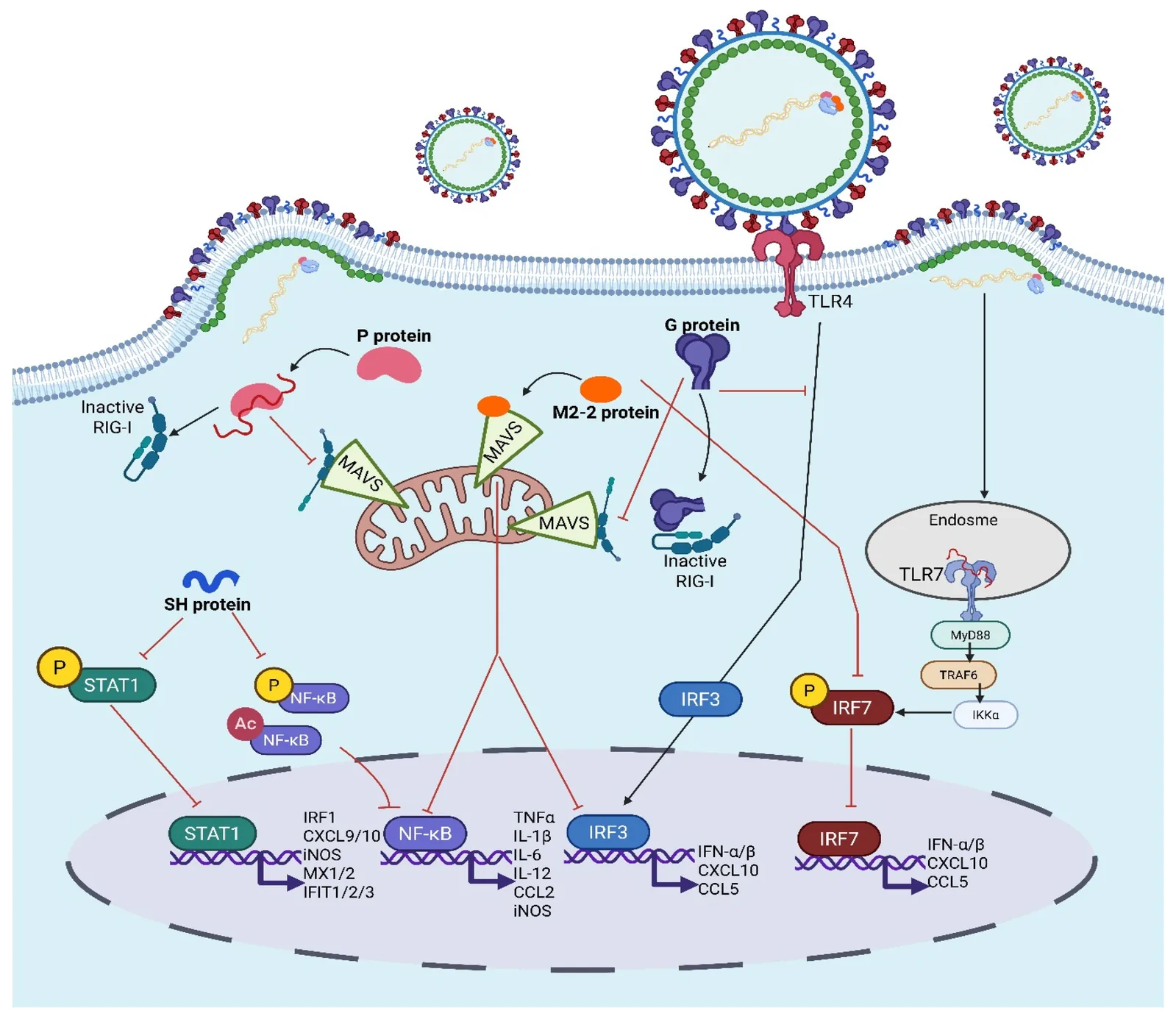
Immune evasion mechanisms of hMPV. Schematic overview of how hMPV proteins interfere with host pattern-recognition receptor (PRR) signaling to block IFN and proinflammatory gene expression. At the plasma membrane, TLR4 and cytoplasmic RIG-I detect viral components and signal through MAVS at the mitochondrial membrane, while endosomal TLR7 signals through the MyD88/TRAF6/IKKα axis. As detailed in Section 5, the viral P, G, M2-2, and SH proteins (red inhibitory lines) each block a distinct step of these cascades: P protein keeps RIG-I in its inactive form; G protein inhibits both TLR4 and RIG-I/MAVS signaling; M2-2 protein binds MAVS and prevents IRF7 phosphorylation downstream of TLR7; and SH protein blocks phosphorylation of STAT1 and phosphorylation/acetylation of NF-κB. Together, these blockades prevent nuclear translocation of STAT1, NF-κB, IRF3, and IRF7, thereby suppressing transcription of their respective target genes, including interferon-stimulated genes (IRF1, CXCL9/10, iNOS, MX1/2, IFIT1/2/3), proinflammatory mediators (TNF-α, IL-1β, IL-6, IL-12, CCL2, iNOS), and type I IFNs and chemokines (IFN-α/β, CXCL10, CCL5).

hMPV, similar to other viruses, can impede IFN signaling; however, the precise processes remain inadequately elucidated. Nonetheless, other viral proteins have been associated with these mechanisms, including the G, SH, and M2-2 proteins [24,47,50,55]. Certain hMPV-A and -B strains contain a P protein that can prevent RIG-I from detecting 5′-triphosphate viral RNA, which in turn stops the synthesis of IFN-I and the development of ISGs [106]. hMPV can stop STAT1 and STAT2 from being activated, both of which are essential transcription factors in the IFN signaling pathway. This weakens the antiviral response [55]. Figure 3 illustrates how interactions between viral proteins and key components of host immune signaling pathways constitute a major mechanism through which hMPV evades the immune response. Zhang et al. conducted a study that demonstrated that IFN-I and IFN-III serve fundamentally distinct functions during hMPV-A infection [107]. They infected genetically modified STAT1/2-deficient (STAT-KO) mice, which lack both type I and type III IFN signaling due to the absence of signal transducers. The absence of STAT1/2 somewhat alleviated the illness severity caused by the pathogenic strain C2-202. STAT-KO animals infected with C2-202 showed less weight loss and were more active than the infected wild-type mice. The lack of IFN signaling also restored C2-202’s ability to replicate in the lung. To ascertain the roles of both IFNs in immunopathology, researchers selectively inhibited the receptors of these IFNs using particular antibodies. Interestingly, inhibition of the type I IFN signaling (via IFNAR) pathway diminished lung pathology and clinical severity without significantly enhancing viral multiplication. This suggests that IFN-I disproportionately influences immunopathology rather than facilitating effective viral control. In contrast, blockage of type III IFN signaling (via IFNLR) did not modify illness severity. However, it reinstated viral replication, underscoring its principal function in antiviral limitation rather than in promoting inflammation [107]. These observations indicate that targeted techniques for managing severe hMPV infection mitigate detrimental inflammation while maintaining antiviral defense.

## 8. Epithelial Cells and Their Role in hMPV Infection and Persistence

The airway epithelium is the principal target of hMPV infection. The identification of genes and pathways triggered by this respiratory virus is crucial for comprehending the pathogenesis of hMPV-induced lung illness [108]. The innate immune response to hMPV begins with airway epithelial cells, alveolar macrophages, and DCs, which identify the virus through pattern recognition receptors (PRRs) such as retinoic acid inducible gene I (RIG-1) and melanoma differentiation-associated 5 (MDA5). This causes the body to produce type I and III IFNs and proinflammatory cytokines [12]. According to a study by Bao et al., A549 cells, a human alveolar basal epithelial cell line derived from a lung adenocarcinoma, release several cytokines, chemokines, and type I IFNs when they are infected with hMPV [108]. Moreover, TLR3 expression is upregulated in epithelial cells (AECs) following hMPV infection; however, suppressing TLR3 expression does not affect hMPV-induced chemokine gene expression in A549 cells [86]. Moreover, the evidence substantiates the hypothesis that hMPV disrupts many PRRs and cell signaling pathways activated by IFN-associated genes. Nevertheless, the processes identified in hMPV differ from those in hRSV, as the latter possesses two non-structural proteins that can inhibit the IFN signaling pathways and the antiviral innate recognition pathways activated by PRRs [25]. However, hMPV has developed strategies to suppress innate antiviral immune responses, such as reducing IFN signaling and impairing dendritic cell function. This slows down or weakens the antiviral defense [109,110].

The viral RNA of hMPV can be detected in respiratory epithelial cells for an extended period, lasting up to 180 days post-infection. These findings indicate that hMPV may have evolved mechanisms enabling its persistence within the host cell, thereby evading viral clearance for a minimum of two months post-infection [111,112]. Marsico et al. elucidated that an hMPV-A strain can endure within A549 cells by obstructing the apoptotic mechanism. This persistence is primarily attributable to the heightened expression of antiapoptotic proteins such as Bcl-2 [113]. hMPV bronchiolitis in early childhood is a significant risk factor for the development of asthma. The virus appears to evade clearance by the immune system and continues to replicate at a low level, which can cause persistent lung inflammation and the development of disease [114].

## 9. Innate Immune Response to hMPV

In pulmonary physiology, safeguarding against pathogenic germs is not exclusively reliant on physical and chemical barriers. Innate immune cells are essential for the response to numerous respiratory viruses owing to their significant diversity, which enables them to preserve homeostasis and facilitate inflammatory processes [115]. Among them, resident immune cells in the respiratory tract, including alveolar macrophages (AMs), innate lymphoid cells (ILCs), and unconventional T lymphocytes, are specialized to offer sustained or protracted defense of this tissue [116]. Numerous investigations connecting hMPV to the innate immune system have shown that C57BL/6 mice lacking natural killer (NK) cells show no reduction in viral titers compared to control mice after infection with the clinical strain hMPV A2 TN/94-49. This contrasts with previous viral infections in which NK cells are essential for viral eradication, indicating that the swift elimination of hMPV may not depend on NK cells [117]. Furthermore, BALB/c mice infected with hMPV exhibited a notable elevation in neutrophil counts in bronchoalveolar lavage (BAL) samples compared to those infected with rhMPV-ΔG [48].

AMs are the most important innate immune cells for the first response to a virus. They perform effector and immunomodulatory roles. Macrophages have significant functional flexibility and polarize into various phenotypes depending on the microenvironment [118]. In vivo investigations have validated the active involvement of AMs in hMPV-induced immunopathogenesis. AMs are a significant source of cytokines such as IL-6, TNF-α, IFN-α/β, CCL4, GM-CSF, and G-CSF, which strengthen the inflammatory response in the lower respiratory tract [119]. The lack of AMs during the initial phase of infection correlated with diminished viral replication, indicating that these macrophages may promote infection rather than hinder it [120]. hMPV has demonstrated the capacity to elicit a vigorous and prolonged type I and III IFNs (IFN α/β and IFN λ) response in human monocyte-derived macrophages and THP-1 cells, accompanied by significant ISG activation [121].

Numerous studies have shown that hMPV infection in the respiratory epithelium triggers a robust chemotactic response through the release of cytokines and chemokines. CCL5/RANTES consistently exerts a substantial influence on this response, recruiting neutrophils and eosinophils, and is also associated with increased asthma symptoms in the lungs [122]. Initial studies in A549 cells and primary alveolar epithelial cultures indicated that hMPV infection induces significant production of IL-8 and CCL5 via a mechanism reliant on the activation of NF-κB and IRFs [108]. These results demonstrated that active viral replication is necessary for the commencement of proinflammatory signaling. Transcriptomic analyses later confirmed that hMPV infection induces a diverse array of CC and CXC chemokines, including CCL5, establishing it as a key mediator of the virus-associated inflammatory response [47]. A further investigation by the same group used a mutant hMPV that did not have the G gene (ΔG) to show that loss of G substantially increased the production of cytokines and chemokines, particularly CCL5. In conclusion, these results demonstrate that this chemokine is not only a hallmark of the epithelial response but also a critical node subject to viral modulation [49]. In addition to observations in the respiratory epithelium, research involving macrophages and animal models has demonstrated that hMPV-A produces chemokines such as CCL5, which induce inflammation. These responses are altered upon interaction of the virus with pre-existing immune cells, particularly macrophages [123]. In a murine model, hMPV-A infection induced a pulmonary cytokine and chemokine profile in BAL fluid, marked by CCL5, resembling the pattern produced by RSV. This emphasizes CCL5 as a significant regulator of respiratory paramyxovirus responses [123]. Kolli and colleagues built on these findings by showing that AMs have a direct function in controlling the production of chemokines in response to infection with the hMPV-A strain. A targeted reduction in AMs led to increased CCL5 synthesis in hMPV-infected rats, indicating that AMs serve not only as chemokine producers but also as regulators of the local inflammatory response. These findings generally corroborate the idea that the epithelium initiates the production of chemokines, while AMs predominantly regulate the magnitude and velocity of pulmonary inflammation resolution [120].

DCs are another type of innate immune cell that is crucial for initiating adaptive responses since they activate lymphocytes [26]. In response to inflammation, primary antigen-presenting cells (APCs), such as DCs and macrophages, are attracted to the infection site [74]. Following hMPV infection, DCs may be activated directly through PRRs or indirectly via proinflammatory cytokines and chemokines secreted by airway epithelial cells or other local immune cells [12,124]. These cells engulf infected cells, viral particles, and debris. Subsequently, phagolysosomes break down viral proteins and present them to CD4^+^ T-helper cells on MHC class II and CD8^+^ cytotoxic T cells on MHC class I. APCs also produce cytokines like IL-12 and TNF-α, which enhance immune system function [74]. The start of the immune response through IFNs, chemokines, cytokines, and the upregulation of costimulatory molecules is essential for the activation of virus-specific CD4^+^ T cells and the development of antiviral defense [12]. The replication of hMPV in DCs can be either productive or abortive, contingent upon the strain and host, as demonstrated in in vitro models utilizing human conventional DCs (cDCs) and mouse bone marrow-derived DCs (BMDCs) [26,27,125]. Different hMPV proteins hinder the operation of dendritic cells and the host’s immune response. The G and SH proteins stop CD4^+^ T cells from becoming active. The P protein prevents RIG-I from sensing IFN-I, which in turn prevents it from being produced. The SH protein prevents STAT1 from being phosphorylated, thereby inhibiting ISG transcription. Numerous PRRs can detect hMPV-associated pathogen-associated molecular patterns (PAMPs), especially within DCs [9,73,126]. These immune evasion methods lead to inadequate immunological responses, virus persistence, and recurrent reinfection. A thorough understanding of how hMPV and DCs interact is necessary for developing effective antiviral drugs and vaccines.

## 10. Adaptive Immune Responses to hMPV

Adaptive immunity is crucial for eradicating viral infections and forming durable immunological memory against a specific pathogen, which facilitates a more rapid response to subsequent exposures [127,128]. The three main types of cells that make up the adaptive immune response are (i) B cells, which produce antibodies; (ii) CD4^+^ T cells, which have different subtypes, such as Th1, Th2, Th17, regulatory T cells (Tregs), and T-follicular helper cells (Tfhs); and (iii) CD8^+^ T cells, which directly kill infected cells in the airways via cytokines and have cytotoxic effector functions [128,129,130]. The adaptive immune response begins in the draining lymph nodes located within the nasopharynx-associated lymphoid tissue (NALT) and bronchus-associated lymphoid tissue (BALT). APCs, primarily conventional DCs, migrate after being activated by respiratory viruses [127]. T cells are crucial in regulating hMPV replication in the lung during both the initial and later stages of hMPV infection. Depletion of the T cell population in mice increased viral replication in the lungs, particularly when CD4^+^ T cells were absent [12,111,131]. As previously stated, APCs display antigens through MHC Class II, thereby activating CD4^+^ T-helper cells. Subsequently, IL-12 causes Th1 polarization, leading to IFN-γ production, which then activates macrophages and boosts antiviral defenses. However, recent studies in neonatal mouse models have shown that this pathway is significantly restricted in early life. Brown et al. showed that, following hMPV-A infection, neonates display a reduction in Th1 differentiation and increased Th2 and regulatory T cell (Treg)-like responses. This inhibition is extrinsic in nature and is mediated by elevated transforming growth factor beta (TGF-β) production by Tregs and inhibitory programmed cell death protein 1 (PD-1) signaling in CD4^+^ cells. Together, these findings reveal that, although T cells are essential for viral control, the neonatal immunoregulatory environment deliberately limits the Th1 response, which could contribute to the greater severity observed in hMPV infections in infants [132]. Likewise, Th2 responses facilitate B cell activation and the synthesis of antibodies, including IgG and IgA. Subsequently, CD4^+^ T-helper cells activate B cells, leading to the production of neutralizing antibodies, such as IgG for systemic immunity and IgA for mucosal protection [12,74].

The function of CD4^+^ T cells in hMPV infections has not been well examined; nevertheless, it has been demonstrated that hMPV can suppress the activation of CD4^+^ T cells in vitro [9,27]. These cells generate a suboptimal IFN-γ response upon activation with hMPV-infected peripheral blood mononuclear cells (PBMCs) in vitro [133]. The observed deterioration of T cell immunity following hMPV infection is likely attributable to the compromised activity of hMPV-infected DCs. In vitro studies have demonstrated that SH and/or G proteins diminish CD4^+^ T cell proliferation in an assay involving co-culture with hMPV-infected dendritic cells [134]. An interesting study by Lamens et al. showed the fundamental role played by hMPV-specific CD4^+^ T cells. Their analysis revealed that hMPV-specific pulmonary T cells peaked on day 10 post-infection and polarized toward a Th1 profile. However, CD4^+^ T cells displayed phenotypic and functional markers of impairment, including co-expression of inhibitory receptors and prolonged PD-1 upregulation. Despite this dysfunction, CD4^+^ T cell depletion in a mouse model of acute infection led to delayed viral clearance and increased PD-1 expression on hMPV-specific CD8^+^ T cells. They thus demonstrated the role of CD4^+^ T cells in viral clearance and in the regulation of CD8^+^ T cell responses [135].

On the other hand, Tregs can suppress effector cell function and proliferation by secreting inhibitory cytokines such as transforming TGF-β and IL-10. Moreover, Tregs can modulate dendritic cell function through the interaction of inhibitory receptors, including cytotoxic T-lymphocyte-associated protein 4 (CTLA-4) and PD-1, with their ligands, CD80/CD86 and PD-L1/2, respectively [136].

Tregs increase and become active in the lungs of mice when they are exposed to hMPV-A [137]. Rogers and colleagues demonstrated that Tregs are crucial during the early phase of infection, not by directly activating DCs or T cells but by maintaining an environment that supports DC and T cell migration and proper priming of antiviral responses. In the absence of Tregs, initial immune activation is limited, polarizing the response toward a Th2 profile. Once the antiviral response is established, however, Tregs become dispensable and can even restrain effector T cell function, potentially compromising viral clearance. Mechanistically, Tregs can reduce the expression of costimulatory molecules such as CD80 and CD86 on DCs, thereby limiting sustained activation of effector T cells [137]. The impact of hMPV-A infection on Tregs was evident in a study involving IL-17-defective mice, which exhibited a deficiency in IL-17 production. Employing IL-17 knockout mice, the study demonstrated that IL-17 deficiency diminishes the infiltration of neutrophils, as well as the quantities of IFN-γ^+^ (Th1) and IL-4^+^ (Th2) T lymphocytes in the lungs of hMPV-infected mice, while increasing the number of Treg cells, compared to uninfected mice. These findings indicate that IL-17 may have a detrimental effect during hMPV infections, as this cytokine may facilitate the development of the proinflammatory environment previously characterized [9,138].

Virus-specific CD8^+^ T cells are capable of selectively eradicating infected cells and are crucial during respiratory virus infections. Tzannou et al. demonstrated the presence of hMPV-reactive T cells in the peripheral blood of both healthy and immunocompromised individuals, albeit at low levels, necessitating prior expansion of these specific T cells. The researchers established a hierarchy of immunodominance based on many donors who exhibited favorable responses to hMPV antigens. Interestingly, the F protein was identified by 97% of the donors, followed by N, M2-1, M, P, L, G, and SH in that order of dominance [139].

Research indicates that infection with the hMPV-A strain produces functional anergy in CD8^+^ T cells, thereby diminishing the efficacy of the antiviral response. For instance, the study conducted by Erickson et al. demonstrates that hMPV infection in mice leads to the impairment of CD8^+^ T cells, marked by diminished IFN-γ production and decreased cytotoxic capacity. This intriguing study shows that CD8^+^ T lymphocytes become exhausted, upregulate PD-1, and subsequently fail to react upon re-stimulation with viral antigens. PD-1 expression is elevated in various settings, including viral infections and malignancies, and is essential for regulating an appropriate immune response to hMPV [140,141]. Following hMPV infection, PD-1-mediated reduction in pulmonary CD8^+^ T cells proceeded swiftly, manifesting by day seven and persisting for several weeks post-viral clearance [141]. Notably, hMPV stops the activation of cytotoxic CD8^+^ T cells, which are crucial for eliminating infected cells [142]. Moreover, hMPV promotes the production of molecules including PD-1 and T-cell immunoglobulin and mucin-domain-containing-3 (Tim-3) on T cells, thereby inhibiting their effector activity. This profile resembles that observed in states of exhaustion or anergy during other chronic viral infections [143]. hMPV-induced anergy in T cells influences both the acute phase of infection and the development of an efficient memory response, facilitating recurrent hMPV infections. This anergy is marked by a diminished immunological response, characterized by inefficient T cell polarization or restricted activation, potentially resulting from low cytokine release levels [144]. Building on these findings, recent work by Parks et al. demonstrated that the severity of respiratory viral infections is further exacerbated in elderly hosts due to the accumulation of terminally exhausted CD8^+^ T cells. These cells showed a T cell transcription factor 1 (TCF1^−^)-negative and TOX (TOX^+^)- and EOMES (EOMES^+^)-positive phenotype with high and sustained PD-1 expression and markedly reduced proliferative and cytotoxic capacity. This terminal exhaustion is age-intrinsic because the researchers proved that transfer of aged CD8^+^ T cells to young hosts does not protect against the virus, whereas young CD8^+^ T cells transferred to elderly hosts partially restore immunity. These results suggest that, in addition to transient PD-1-mediated dysfunction, the presence of terminally exhausted T cells in older individuals contributes to more severe disease outcomes [145].

The production of virus-specific antibodies by B lymphocytes is a key component of the immune response against viral infections. These antibodies can neutralize or opsonize virions, facilitating their inactivation, and contribute to the destruction of infected cells. Limiting the spread of infectious virions to neighboring cells is crucial for controlling viral infection. For example, hMPV infection targets antigens like the fusion (F) protein, which confers long-term immunity [74]. Mechanistically, neutralizing antibodies against the F protein are thought to act primarily by blocking the conformational rearrangement that occurs during membrane fusion, thereby preventing the metastable prefusion conformation of F from refolding into the postfusion state and consequently preventing insertion of the fusion peptide into the host cell membrane. Since F binding has also been linked to interactions with heparan sulfate and, potentially, with the α5β1 integrin through an RGD motif, antibodies targeting these binding regions could additionally interfere with viral attachment prior to membrane fusion [65,146].

In contrast to the F protein, the attachment glycoprotein G does not appear to represent a major neutralizing or protective antigen of hMPV. A study evaluating the individual contributions of the surface glycoproteins F, G, and SH to the induction of neutralizing antibodies and protective immunity in hamsters demonstrated that F was highly immunogenic and protective, whereas G and SH were only weakly or minimally immunogenic and protective, respectively. The authors concluded that hMPV G does not constitute a major antigen for neutralization or protection [147]. This finding was independently confirmed by immunization of cotton rats with a soluble recombinant G ectodomain, which elicited high serum antibody titers against G but failed to induce neutralizing antibodies or confer protection against viral challenge, a feature that the authors described as unusual compared with the attachment glycoproteins of other human paramyxoviruses [148]. The absence of neutralizing activity against G has been attributed to the masking of potential neutralizing epitopes by its extensive glycosylation, together with the inability of recombinant G ectodomain constructs to recapitulate native conformational epitopes. Interestingly, immunization with G fused to carrier proteins (CTB or CRM197) promoted viral clearance in mice despite the absence of neutralizing antibodies, suggesting a potential contribution of antibody-dependent, non-neutralizing protective mechanisms that warrants further investigation [149].

Following the primary infection, memory B cells are generated, facilitating a rapid response to repeated infections by the same virus [150] and ensuring long-term immunity to hMPV [74]. A negative relationship exists between anti-hMPV antibody levels and vulnerability to hMPV infection [151]. It is crucial to acknowledge that the hMPV-specific antibody response is insufficient for viral clearance, allowing hMPV to survive in the lungs despite the presence of neutralizing antibodies [111,112].

**Figure 4:**
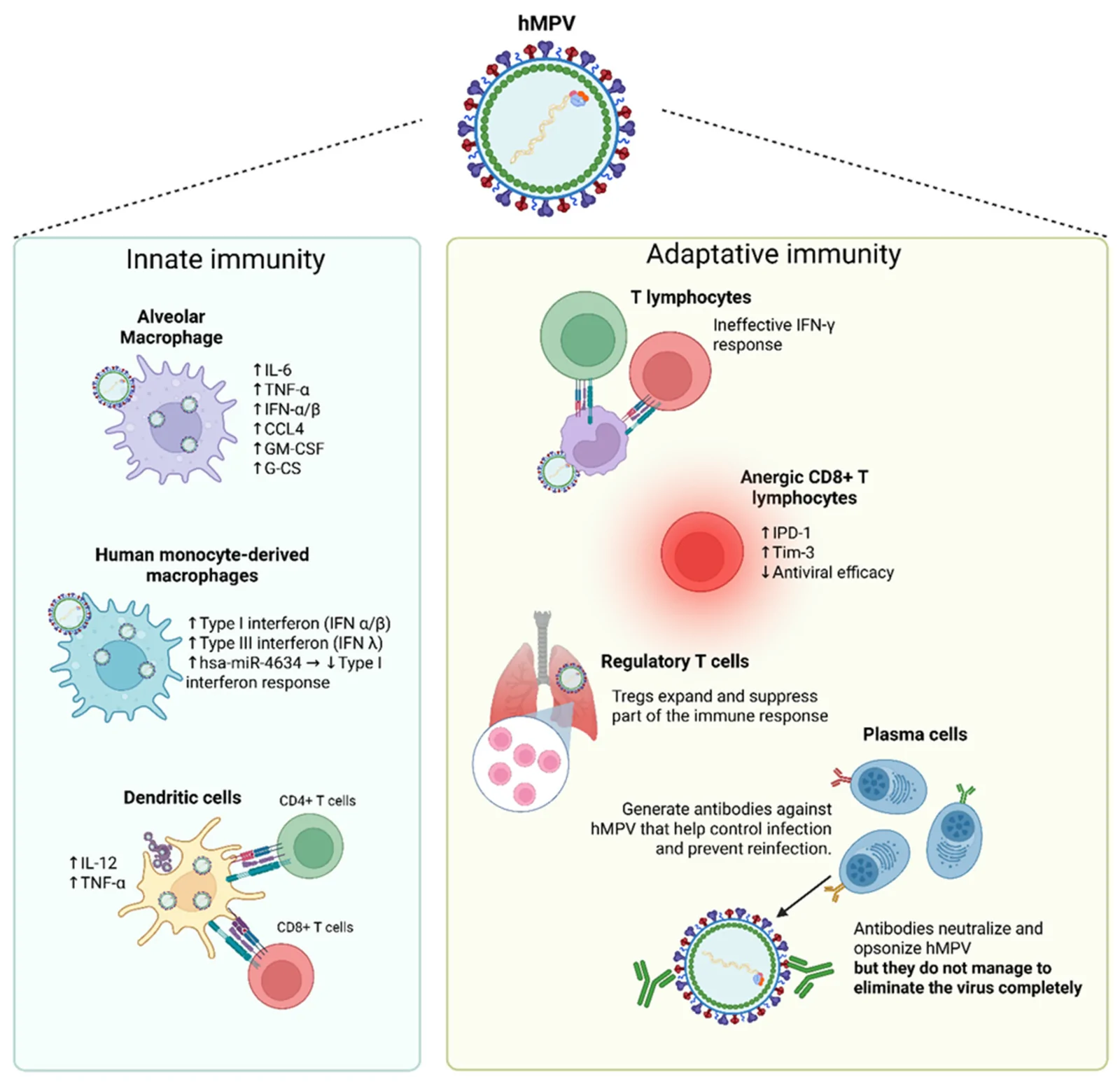
Innate and adaptive immune responses to hMPV. Infection with hMPV is initially sensed by the innate immune system through macrophages and dendritic cells, which produce proinflammatory cytokines and chemokines that modulate T cell activation. Although antigen presentation by infected DCs can stimulate CD4^+^ and CD8^+^ T cells, hMPV elicits only weak development of immune memory. Consequently, the adaptive immune system’s capacity to provide long-lasting protection is limited due to reduced efficiency of CD4^+^ and CD8^+^ T cells, prolonged immunosuppression mediated by regulatory T cells, and persistence of the virus in the lungs. Notably, this viral persistence occurs even in the presence of neutralizing antibodies generated after primary infection, derived from memory B cells.

T and B cells work together to ensure the immune system responds strongly and accurately to hMPV, helping to control and eradicate the infection. In healthy adults, adaptive immunity is generally effective; however, in infants, older adults, and immunosuppressed individuals, the adaptive response is less efficient, favoring viral persistence and increasing the risk of severe disease [152,153,154]. Figure 4 shows how hMPV interacts with innate and adaptive immune systems. This demonstrates a complex immune evasion strategy that makes it difficult for hosts to develop long-term tolerance. While hMPV infection elicits cellular responses mediated by CD4^+^ and CD8^+^ T cells, as well as humoral responses via neutralizing antibodies, these defenses are typically transient and insufficient. This allows for multiple reinfections throughout life. The virus impairs antigen presentation, reduces type I interferon responses, and shifts immune polarization toward less protective Th2 or Th17 profiles. Furthermore, the immune system’s memory is limited, making long-term protection difficult.

## 11. Conclusions

Human metapneumovirus (hMPV) is a significant public health concern due to its propensity to cause recurring respiratory infections and its ability to evade both innate and adaptive immune responses. Even though it was discovered more than twenty years ago, its immune evasion mechanisms still make it difficult to develop effective treatments and long-lasting vaccines. This review delineated and integrated the primary mechanisms by which hMPV interferes with host sensing and signaling networks. These include suppressing the generation of type I IFNs, impairing the development of dendritic cells, inhibiting T cell activation, and altering microRNAs that help the virus remain in the body. In addition, the virus’s interaction with regulatory molecules like PD-1 and Tim-3 puts the immune system in a state of immunological anergy, making it difficult for the immune system to respond effectively and for an extended period. A comprehensive understanding of the immune evasion strategies employed by hMPV is essential not only for guiding therapeutic and immunological approaches but also for highlighting areas that require further investigation. In particular, the roles of specific immune cell subsets such as innate lymphoid cells, DC subpopulations, and B cell memory formation remain incompletely understood. Further research into these mechanisms could inform the rational design of live-attenuated vaccines and antiviral agents that effectively block hMPV immune evasion, enhance protective immunity, and reduce the risk of reinfections. Understanding these processes is especially important for developing strategies to improve infection management in vulnerable populations, such as infants and older adults.

## Figures and Tables

**Table 1:** Summary of human metapneumovirus proteins and their functions.

Gene	Protein(s)	Function(s)
*N*	Nucleoprotein	Nucleocapsid formation, regulates genome replication
*P*	Phosphoprotein	Polymerase factor, may protect the virus from RIG-I
*M*	Matrix protein	Regulates viral assembly and budding
*F*	Fusion protein	Cell entry
*M2*	M2.1 and M2.2 proteins	Polymerase antitermination factor (M2.1) inhibits the mitochondrial antiviral signaling protein (MAVS) and regulates the genome replication (M2.2)
*SH*	Small hydrophobic protein	Innate immune inhibitor, viroporin, modulates fusogenicity of F
*G*	Attachment glycoprotein	Host cell attachment, RIG-I inhibition
*L*	Large polymerase protein	Polymerase factor

## Data Availability

No new data were created or analyzed in this study. Data sharing is not applicable to this article.
